# Lactate fluxes mediated by the monocarboxylate transporter-1 are key determinants of the metabolic activity of beige adipocytes

**DOI:** 10.1074/jbc.RA120.016303

**Published:** 2020-12-06

**Authors:** Damien Lagarde, Yannick Jeanson, Corinne Barreau, Cedric Moro, Lindsay Peyriga, Edern Cahoreau, Christophe Guissard, Emmanuelle Arnaud, Anne Galinier, Anne-Karine Bouzier-Sore, Luc Pellerin, Edward T. Chouchani, Luc Pénicaud, Isabelle Ader, Jean-Charles Portais, Louis Casteilla, Audrey Carrière

**Affiliations:** 1 STROMALab, Université de Toulouse, CNRS ERL5311, EFS, INP-ENVT, INSERM U1031, Université Paul Sabatier, Toulouse, France; 2 Institut RESTORE, UMR 1301 INSERM, 5070 CNRS, Université Paul Sabatier, Toulouse, France; 3 Institute of Metabolic and Cardiovascular Diseases, INSERM UMR1048, Paul Sabatier University, Toulouse, France; 4 Toulouse Biotechnology Institute TBI - INSA de Toulouse INSA/CNRS 5504 - UMR INSA/INRA 7924, Toulouse, France; 5 MetaboHUB-MetaToul, National Infrastructure of Metabolomics and Fluxomics, Toulouse, France; 6 Institut Fédératif de Biologie, CHU Purpan, Toulouse, France; 7 Université de Bordeaux, CNRS, CRMSB, UMR 5536, Bordeaux, France; 8 INSERM U1082, Université de Poitiers, Poitiers Cedex, France; 9 Department of Cancer Biology, Dana-Farber Cancer Institute, Boston, Massachusetts, USA; 10 Department of Cell Biology, Harvard Medical School, Boston, Massachusetts, USA

**Keywords:** beige adipocytes, lactate fluxes, monocarboxylate transporters, uncoupling protein 1, glycolysis, ANR, Agence Nationale pour la Recherche, AZD, AZD3965, BAT, brown adipose tissue, CL, CL316.243, LCM, laser capture microdissection, MCTs, monocarboxylate transporters, UCP1, uncoupling protein 1

## Abstract

Activation of energy-dissipating brown/beige adipocytes represents an attractive therapeutic strategy against metabolic disorders. While lactate is known to induce beiging through the regulation of *Ucp1* gene expression, the role of lactate transporters on beige adipocytes' ongoing metabolic activity remains poorly understood. To explore the function of the lactate-transporting monocarboxylate transporters (MCTs), we used a combination of primary cell culture studies, ^13^C isotopic tracing, laser microdissection experiments, and *in situ* immunofluorescence of murine adipose fat pads. Dissecting white adipose tissue heterogeneity revealed that the MCT1 is expressed in inducible beige adipocytes as the emergence of uncoupling protein 1 after cold exposure was restricted to a subpopulation of MCT1-expressing adipocytes suggesting MCT1 as a marker of inducible beige adipocytes. We also observed that MCT1 mediates bidirectional and simultaneous inward and outward lactate fluxes, which were required for efficient utilization of glucose by beige adipocytes activated by the canonical β3-adrenergic signaling pathway. Finally, we demonstrated that significant lactate import through MCT1 occurs even when glucose is not limiting, which feeds the oxidative metabolism of beige adipocytes. These data highlight the key role of lactate fluxes in finely tuning the metabolic activity of beige adipocytes according to extracellular metabolic conditions and reinforce the emerging role of lactate metabolism in the control of energy homeostasis.

Thermogenic brown and beige adipose tissues increase systemic energy expenditure and represent putative targets to cure obesity and related metabolic diseases including type II diabetes (1, 2). The energy-dissipating capacity of brown adipocytes is due to a high mitochondrial content and the expression of uncoupling protein 1 (UCP1) inside the mitochondrial inner membrane, which enables heat production through acceleration of the mitochondrial electron transport chain. Although sharing phenotypic, metabolic, and functional similarities with brown adipocytes, multilocular adipocytes expressing UCP1 interspaced within white adipose tissue (3, 4, 5) are distinct cells, with specific molecular expression profiles and different developmental origins. The number of these so-called beige adipocytes sharply increases during cold exposure through a process known as beiging, particularly in the murine inguinal fat pad, while the perigonadal depot is refractory to beiging (6). We recently highlighted the structural heterogeneity of the inguinal fat pad and localized cold-induced beige adipocytes in the core of the depot, a region defined by the tissue autofluorescence signal (7) and constituted by interconnected and complex 3D polylobular entities (8). Different studies suggest that, depending on the nature or the length of the stimulation of the fat pad, cold/β-adrenergic signaling promotes *de novo* beige fat differentiation and/or induction of UCP1 in mature adipocytes (9, 10, 11, 12). Besides the therapeutic perspectives associated with the beiging-dependent remodeling of adipose tissues, the mechanisms regulating beige adipocyte metabolic activity still remain incompletely understood.

It has been recently demonstrated that beige adipocytes appear in different physiopathological conditions, including cancer-associated cachexia (13, 14), intermittent fasting (15), or physical exercise (16), suggesting function(s) that are distinct from thermogenesis (17). We recently described that lactate, a metabolite produced when the glycolytic production of pyruvate exceeds mitochondrial oxidative capacities and acting as a redox substrate and signaling metabolite (18, 19), is a strong inducer of UCP1 expression in adipocytes (20). This occurs through intracellular redox modifications subsequent to its transport. The regulation of UCP1 expression by lactate has been confirmed by others (21, 22). This mechanism might be part of a redox regulatory and adaptive loop (17, 23) where a high redox (NADH/NAD^+^) pressure drives UCP1 expression, just as mitochondrial reactive oxygen species positively control UCP1 protein activity (24). Lactate also stimulates fibroblast growth factor-21 expression and release by beige adipocytes (25), independently of the redox state, highlighting the diversity of signaling mechanisms responsive to lactate in these cells (23). Among several members of the proton-linked monocarboxylate transporters (MCTs) family that transport lactate, pyruvate, and ketone bodies (26, 27), the MCT1 isoform is known to be expressed by several oxidative tissues, including heart, muscle, and brown adipose tissue (BAT) (28, 29, 30, 31, 32). However, the expression of MCTs by beige adipocytes and their role in their metabolic activity remain to be elucidated to efficiently recruit and/or activate them.

Herein, we report that the MCT1 protein is a marker of inducible beige adipocytes. MCT1 is expressed at the plasma membrane of a subset of adipocytes present in the core region of the inguinal fat pad. These fat cells possess a beige adipocyte gene signature at 21 °C and express the UCP1 protein after short-term cold exposure. Using isotopic labeling experiments, we identified MCT1 as a key transporter mediating simultaneous outward and inward lactate fluxes in beige adipocytes and as a critical target and mediator of the β3 adrenergic signaling. MCT1-dependent lactate fluxes are critical for both glycolysis and oxidative metabolism of beige adipocytes, finely tuning their metabolism according to the extracellular metabolic conditions.

## Results

### Dissecting white adipose tissue heterogeneity reveals a tight correlation between Mct1 and thermogenic gene expression

**Figure 1:**
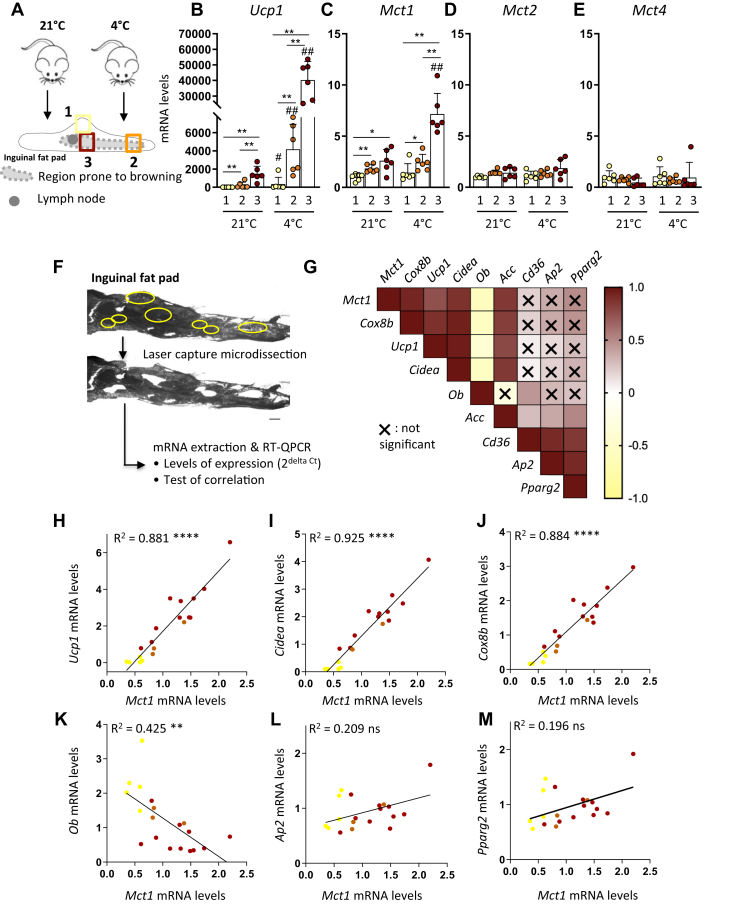
**Tight correlation between *Mct1* expression and beige adipocytes markers in subcutaneous white adipose tissue.***A*–*E*, mice were housed at 21 °C or exposed to 4 °C during 48 h, and gene expression was assessed in selected areas. *A*, schematic representation of the inguinal fat pad and localization of the three dissected areas, in the periphery (region 1), in the core of the tissue lying at the extremity (region 2), and closed to the lymph node (region 3). *B*–*E*, mRNA expression (fold change compared with the region 1 at 21 °C [2^−ΔΔCt^]) of *Ucp1* (*B*), *Mct1* (*C*), *Mct2* (*D*), and *Mct4* (*E*), in regions 1, 2, and 3 (n = 6 mice per group of three independent experiments). *F*–*M*, laser microdissection experiments on inguinal fat pads of 21 °C-housed mice. Gene expression of *Mct1*, *Cox8b*, *Ucp1*, *Cidea*, *Ob*, *Acc*, *Cd36*, *Ap2*, and *Pparg2* was assessed in each microdissected cluster of cells. *F*, picture of an inguinal fat pad section before and after laser microdissection. The *yellow circles* represent the selected areas for microdissection. The scale bar represents 500 μm. *G*, heat map showing positive (*brown*) and negative (*yellow*) correlations between *Mct1*, *Cox8b*, *Ucp1*, *Cidea*, *Ob*, *Acc*, *Cd36*, *Ap2*, and *Pparg2* mRNA expression. X means no significant correlations at the 0.05 significance level. *H*–*M*, correlation graphs between *Mct1* and *Ucp1* (*H*), *Cidea* (*I*), *Cox8b* (*J*), *Ob* (*K*), *Ap2* (*L*), and *Pparg2* (*M*) mRNA expression. Each dot represents a microdissected area. The color of the dot represents the region where the cluster of cells has been dissected (see *A* for color code) (n = 19 microdissected areas from three independent experiments). Data are represented as mean ± SD. Two-tailed nonparametric Mann–Whitney tests. ∗*p* < 0.05, ∗∗*p* < 0.01, ∗∗∗*p* < 0.001 between regions, #*p* < 0.05, and ##*p* < 0.01 between 21 °C and 4 °C for the same region. Pearson's test for simple linear regressions. ∗∗*p* < 0.01, ∗∗∗∗*p* < 0.0001.

Because of the heterogeneity of the beiging-sensitive inguinal fat pad (7, 8), we analyzed mRNA levels of the different *Mct* isoforms described as lactate transporters (26, 27), *i.e.*, *Mct1*, *Mct2*, *Mct3*, and *Mct4*, in specific regions with different beiging abilities (Fig. 1*A*). The spatial heterogeneity of this depot was highlighted by the gradient in *Ucp1* expression, with lowest mRNA levels at the periphery (region 1), intermediate levels in the core of the tissue lying the extremity (region 2), and highest levels close to the lymph node (region 3) (Fig. 1*B*). This gradient was observed in mice housed at 21 °C as well as after 48 h of cold exposure (Fig. 1*B*). *Mct1* expression displayed the same pattern as *Ucp1*, as *Mct1* mRNA levels also increased from regions 1 to 3 and were significantly upregulated following cold exposure, specifically in the region close to the lymph node that exhibited the highest levels of *Ucp1* (Fig. 1*C*). This gene expression profile is specific to *Mct1* as no significant difference was observed regarding *Mct2* or *Mct4* expression, irrespective of the different fat pad regions or in response to cold exposure (Fig. 1, *D*–*E*). *Mct3* was not detected, in accordance with its exclusive expression in retinal cells and choroid plexus (26, 27).

To analyze gene expression with higher precision in this very heterogeneous tissue, we analyzed *Mct1* expression in clusters of cells dissected by laser capture microdissection (LCM; Fig. 1*F*), in the three regions exhibiting different levels of *Mct1* expression, at 21 °C. These experiments revealed a tight and significant positive correlation between *Mct1* and *Ucp1* mRNA levels (*R* = 0.881; *p* < 0.0001; Fig. 1, *G*–*H*) and between *Mct1* and additional thermogenic markers such as *Cidea* and *Cox8b* (*R* = 0.925 and *R* = 0.884, respectively; *p* < 0.0001; Fig. 1, *G*, *I*, and *J*). Conversely, *Mct1* mRNA levels were negatively correlated with leptin (*Ob*) expression (*R* = 0.425; *p* < 0.01; Fig. 1, *G* and *K*), known to be enriched in white adipocytes (33). No significant correlation was found between *Mct1* and other adipogenic genes including *Ap2* and *Pparg2* (Fig. 1, *G*, *L*, and *M*). Thus, the fine dissection of the cellular heterogeneity within the subcutaneous white fat pad highlighted *Mct1* expression as tightly and positively correlated with the gene signature of beige adipocytes.

### MCT1 is expressed by cold-inducible beige adipocytes

**Figure 2:**
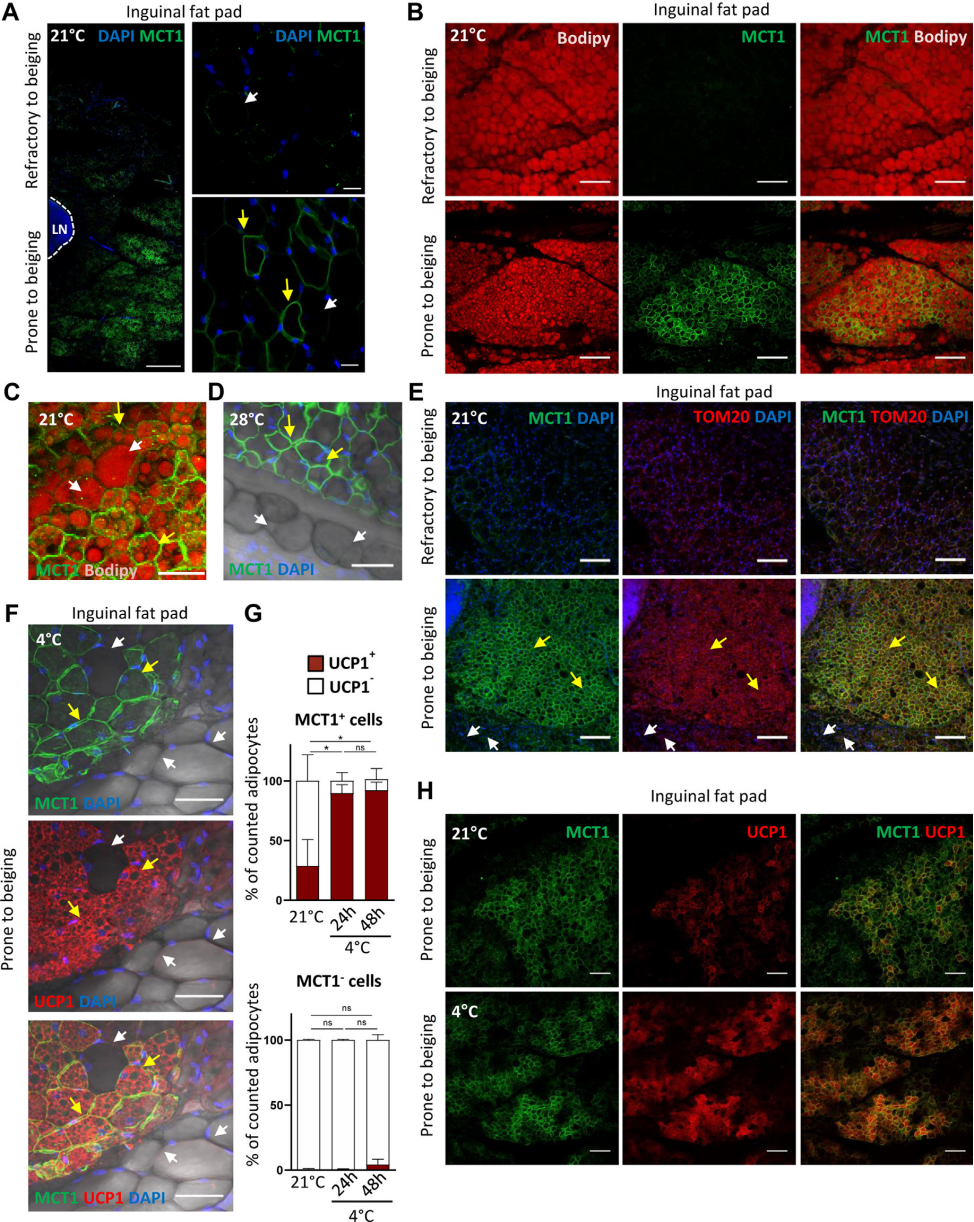
**MCT1 is a marker of cold-inducible beige adipocytes.***A*–*F* and *H*, representative confocal images of 300 μm sections from inguinal fat pad isolated from 21 °C-housed mice (*A*, *B*, *C*, *E*, and *H*), mice acclimated to thermoneutrality (28 °C) (*D*), exposed to cold during 7 days (4 °C) (*F*) or exposed to cold during 2 days (4 °C) (*H*), stained with antibodies recognizing MCT1 (*green*) (*A*–*F* and *H*), costained with the BODIPY lipid probes (*red*) (*B*–*C*), the mitochondrial marker TOM20 (*red*) (*E*), and UCP1 (*red*) (*F* and *H*). Nuclei were stained with DAPI (*blue*). *A*, *C, D*, and *E*, *yellow arrows* represent MCT1^+^ adipocytes and *white arrows* represent MCT1^−^adipocytes. The scale bar represents 500 μM (*A*, *left*), 20 μM (*A*, *right*), 150 μM (*B*), 100 μm (*E* and *H*), and 50 μM (*C*–*D*). *F*, two adjacent but highly different clusters of cells in the inguinal fat pad of a 4 °C-exposed mice are observed: a cluster containing MCT1^+^ UCP1^+^ adipocytes at the *top-left* (*yellow arrows*) and a cluster lacking both MCT1 and UCP1 protein expression (MCT1^−^UCP1^−^) at the bottom right (*white arrows*). The scale bar represents 50 μM. *G*, quantification of the percentage of UCP1^+^ and UCP1^−^ adipocytes in MCT1^+^ (*top panel*) and MCT1^−^ (*bottom panel*) cells in inguinal fat pad of mice housed at 21 °C or exposed to cold (4 °C) for 24 or 48 h (n = 4 mice; for each mice, the average of four images per tissue was performed for each condition). Data are represented as mean ± SD. Two-tailed nonparametric Mann–Whitney tests. ∗*p* < 0.05. DAPI, 4′,6-diamidino-2-phenylindole; MCT1, monocarboxylate transporter 1; UCP1, uncoupling protein 1.

In agreement with *Mct1* mRNA expression patterns, immunofluorescence experiments performed on the whole inguinal fat pad revealed the existence of a gradient in MCT1 protein levels, from the periphery to the core of the tissue (Fig. 2*A*), in 21 °C-housed animals. MCT1 was detected at the plasma membrane of adipocytes, primarily in large clusters in the region prone to beiging close to the lymph node (Fig. 2*A*). In contrast, a very faint signal was detected at the periphery, which is highly refractory to beiging (Fig. 2*A*). In addition, heterogeneous MCT1 protein expression was also observed within the region prone to beiging itself because MCT1^−^ and MCT1^+^ adjacent adipocytes (*white* and *yellow arrows*, respectively; Fig. 2*A*) were identified. MCT1^+^ adipocytes display several small lipid droplets in their cytoplasm and exhibit a paucilocular phenotype (as described (9)), in contrast to unilocular MCT1^−^ adipocytes (*yellow* and *white arrows*, respectively; Fig. 2, *B*–*C*). The same heterogeneous MCT1 staining was observed in the inguinal fat pad of mice acclimated at 28 °C (Fig. 2*D*). Analysis of the outer mitochondrial membrane protein TOM20 indicated that MCT1^+^ adipocytes exhibited a high abundance of mitochondria (Fig. 2*E*; *yellow arrows*), in contrast to MCT1^−^ adipocytes, which very weakly express TOM20 (Fig. 2*E*; *white arrows*). TOM20 is almost undetectable in MCT1^−^ adipocytes of the region of the inguinal fat pad, which is refractory to beiging (Fig. 2*E*; *top panels*). Together, these results reveal a high MCT1 expression in a subset of mature adipocytes gathered in large clusters in the core region of the inguinal pad of mice housed at 28 °C and 21 °C, which may exhibit distinct oxidative capacities.

We next analyzed the expression of the UCP1 protein, at 21 °C and after cold exposure, and quantified in both MCT1^+^ and MCT1^−^ adipocyte subpopulations the percentage of UCP1^+^ and UCP1^−^ cells. These experiments revealed that UCP1 was detected exclusively in the subpopulation of adipocytes expressing MCT1 (Fig. 2, *F*–H). Interestingly, while only 29 ± 11% of MCT1^+^ adipocytes expressed the UCP1 protein at 21 °C, almost all of them turn UCP1^+^ after cold exposure (90 ± 4% and 92 ± 3% of MCT1^+^ adipocytes after 24 and 48 h of cold exposure, respectively, Fig. 2*G*, *top panel*), suggesting that MCT1 is expressed by cold-inducible beige adipocytes. This is reinforced by the fact that the percentage of UCP1^+^ adipocytes in the MCT1^−^ fraction was negligible and did not significantly increase after cold exposure (0.5 ± 0.3 to 4 ± 2%; Fig. 2*G*, *bottom panel*). In conclusion, in the inguinal fat pad, all the paucilocular/multilocular mitochondrial-enriched adipocytes do express MCT1, and the majority of MCT1^+^ adipocytes express UCP1 after cold exposure.

**Figure 3:**
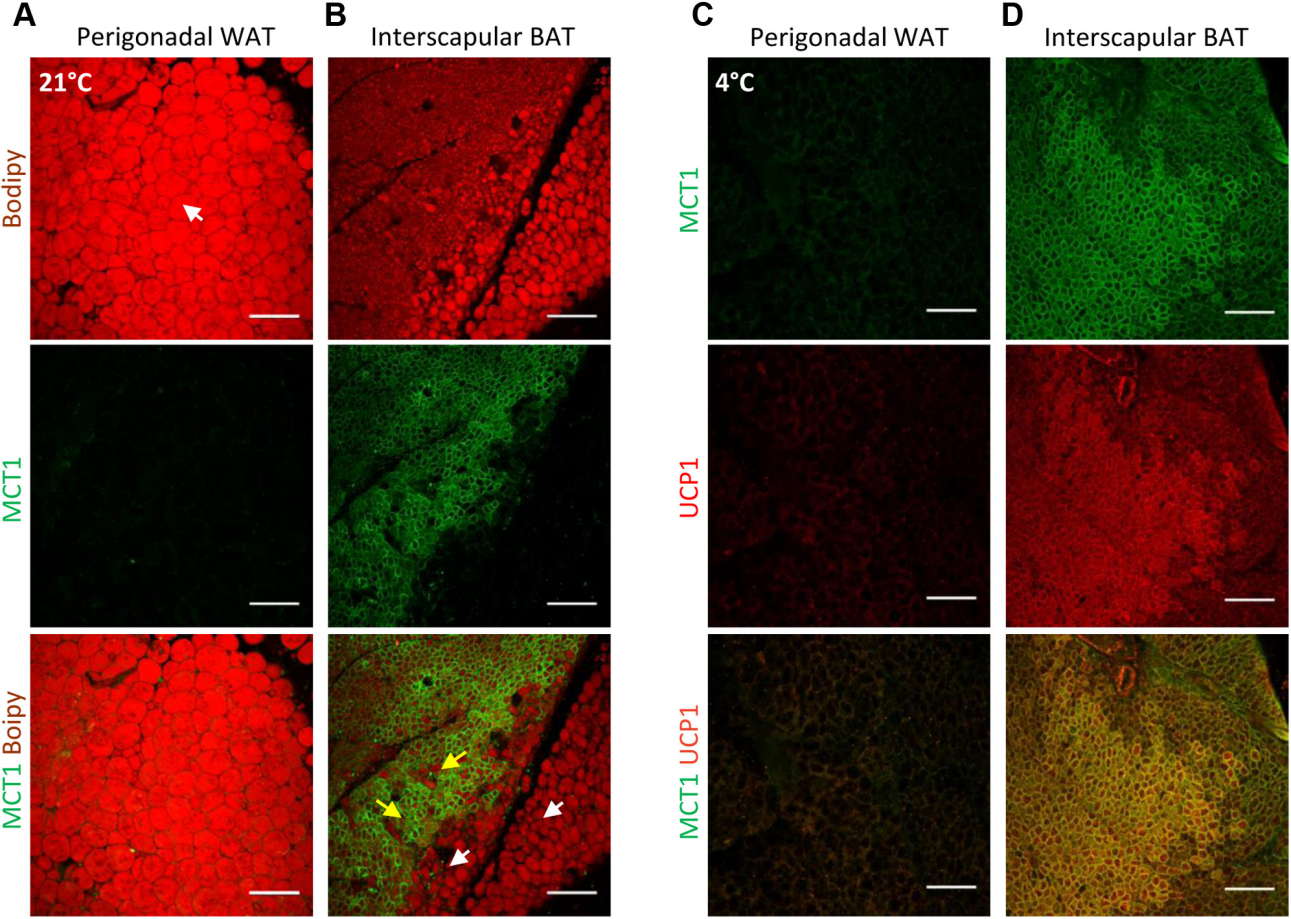
**Expression of MCT1 and UCP1 in perigonadal white adipose tissue and interscapular brown adipose tissue.***A* and *C*, representative confocal images of 300 μm sections from perigonadal white fat pad isolated from 21° C (*A*) or 4° C (*C*) housed mice. *B* and *D*, representative confocal images of 300 μm sections from interscapular BAT isolated from 21° C (*B*) or (*D*) 4 °C housed mice, stained with antibodies recognizing MCT1 (*green*) and costained with the (*A*–*B*) BODIPY lipid probe (*red*) or (*C*–*D*) UCP1 (*red*). The scale bar represents 150 μm. BAT, brown adipose tissue; MCT1, monocarboxylate transporter 1; UCP1, uncoupling protein 1; WAT, white adipose tissue;

In agreement with MCT1 as a *bona fide* marker of inducible beige adipocytes, we could not detect any MCT1 protein in adipocytes from the perigonadal depot at 21 °C or after 4 °C exposure (Fig. 3, *A* and *C*), which is refractory to cold-induced beiging (6). As previously reported (29, 30, 31, 32), we detected high expression of MCT1 in BAT (Fig. 3*B* [*yellow arrows*], Fig. 3*D*). Indeed, all multilocular brown adipocytes expressing UCP1 are MCT1^+^ (Fig. 3*D*). Note that the MCT1 protein was not detected in white adipocytes surrounding BAT (Fig. 3*B*; *white arrows*).

Together, these findings reveal that MCT1 is expressed in classical brown adipocytes and white adipocytes susceptible to beiging remodeling but not in white adipocytes refractory to this process.

### MCT1 is a transcriptional target of the β3-adrenergic pathway and not required for its signaling effect on Ucp1 expression

**Figure 4:**
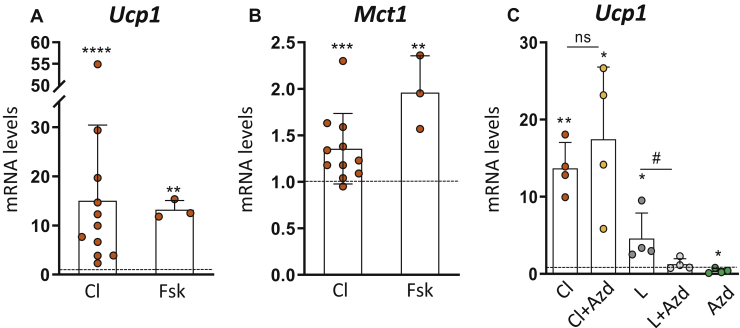
**MCT1 is a transcriptional target of the β3-adrenergic pathway and is not required for its signaling effect on *Ucp1* expression.***A*–*B*, mRNA expression (fold change compared to control cells [*dotted line*] [2^−ΔΔCt^]) of (*A*) *Ucp1* and (*B*) *Mct1* in primary inguinal differentiated adipocytes treated with 10 nM CL316.243 (Cl) for 24 h or 10 μM forskolin (Fsk) for 6 h (n = 3–11 independent primary cultures). *C*, mRNA expression (fold change compared to control cells [*dotted line*] [2^−ΔΔCt^]) of *Ucp1* in primary inguinal differentiated adipocytes treated with 10 nM CL316.243 (Cl), 25 mM sodium-lactate (L), with or without 50 nM AZD3965 (AZD) for 24 h (n = 4 independent primary cultures). Data are represented as mean ± SD. Two-tailed nonparametric Mann–Whitney tests. ∗*p* < 0.05, ∗∗*p* < 0.01, and ∗∗∗*p* < 0.001.

To determine whether the cold-induced *Mct1* expression observed *in vivo* (Fig. 1*C*) could be due to the β3-adrenergic signaling pathway, we treated primary differentiated adipocytes with the β3-adrenergic receptor agonist CL316.243 (CL). We found that CL, which upregulated *Ucp1* expression as expected (Fig. 4*A*), also increased *Mct1* expression in primary differentiated adipocytes (Fig. 4*B*). As the same effect was observed with the cAMP-rising agent forskolin (Fig. 4, *A*–*B*), we concluded that *Mct1* expression was regulated by a cAMP-dependent signaling, further suggesting its functional role in cold-induced beige adipocytes. To study the involvement of MCT1 in β3-adrenergic regulation of *Ucp1* expression, we tested the effect of AZD3965 (AZD), an established MCT1 inhibitor (34, 35, 36, 37). Treatment with 50 nM AZD did not hamper CL-induced *Ucp1* expression (even at higher doses, data not shown) suggesting that MCT1 was not involved in the signaling cascade linking the β3-adrenergic receptor to the regulation of *Ucp1* expression (Fig. 4*C*). We then investigated the effect of AZD on lactate-induced *Ucp1* expression, using sodium-L-lactate (and not lactic acid to avoid pH changes) at a concentration of 25 mM, known to give rise to the maximal induction of *Ucp1* (as shown by the dose-dependent effects reported (20)). We found that AZD abrogated lactate-induced *Ucp1* expression (Fig. 4*C*), confirming previous data obtained with other MCT inhibitors (20), and further highlighting the role of intracellular lactate on the regulation of *Ucp1* expression. These data clearly indicate the existence of two independent signaling pathways regulating *Ucp1* expression, one mediated by MCT1/lactate and the other mediated by β3 adrenergic receptor. Altogether these data reveal that MCT1 is a transcriptional target of the β3 adrenergic pathway and is not involved in the regulation of *Ucp1* expression by β3 agonists.

### MCT1-dependent lactate fluxes are required for β3-adrenergic activation of glycolysis in beige adipocytes

**Figure 5:**
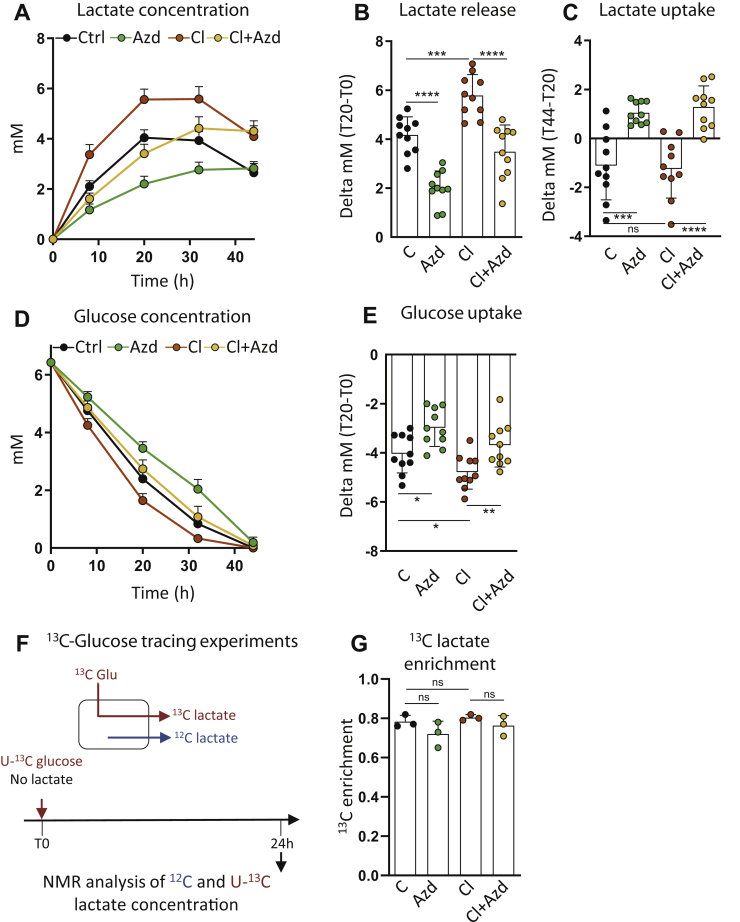
**MCT1-dependent lactate fluxes mediate β3-adrenergic activation of glycolysis in beige adipocytes.***A* and *D*, time-course variation of (*A*) lactate and (*D*) glucose concentration in the supernatant of primary inguinal differentiated adipocytes treated with 50 nM AZD3965 (AZD), 10 nM CL316.243 (Cl), or both, in a glucose-containing medium. *B*, *C*, and *E*, net production of lactate between 0 and 20 h of culture (*B*), net consumption of lactate between 20 and 44 h of culture (*C*) and glucose consumption between 0 and 20 h of culture (*E*) by primary inguinal differentiated adipocytes treated with 50 nM AZD3965 (AZD), 10 nM CL316.243 (Cl), or both, in a glucose-containing medium. (n = 10 independent primary cultures). *F*, adipocytes were incubated in U-^13^C glucose medium for 24 h and ^13^C lactate enrichment (*G*) in cell supernatants were analyzed by NMR after 24 h of culture. (n = 3 independent primary cultures). Data are represented as mean ± SD. Two-tailed nonparametric Mann–Whitney tests. ∗*p* < 0.05, ∗∗*p* < 0.01, ∗∗∗*p* < 0.001, and ∗∗∗∗*p* < 0.0001.

We next investigated the role of MCT1 in the metabolism of beige adipocytes. The effect of AZD on lactate metabolism was first studied by monitoring lactate content in the supernatant of primary adipocytes. Time-course experiments highlighted a biphasic curve where adipocytes exhibited a first phase of net lactate release and a second phase of net lactate consumption after 32 h in culture (Fig. 5*A*). Lactate release was significantly reduced by AZD (Fig. 5, *A*–*B*), showing the primary role of MCT1 in lactate export. The residual export is probably because of other isoforms such as MCT4 (20, 38) although we could not detect its protein expression in adipocytes *in vivo* (data not shown). Strikingly, inhibiting MCT1 abrogated lactate consumption by primary differentiated adipocytes (Fig. 5, *A* and *C*), as found in cell lines (38). As expected (39, 40, 41), lactate production was increased upon treatment with the β3-adrenergic receptor agonist, and we found that MCT1 activity was required for this effect (Fig. 5, *A*–*B*). Of note, the rate of lactate consumption was comparable in CL-treated cells compared to control conditions (Fig. 5, *A* and *C*).

Because lactate is mostly derived from glucose catabolism, we next analyzed the consequences of MCT1 inhibition on glucose consumption. We found that inhibiting lactate transport strongly reduced the glycolytic flux in beige adipocytes as blocking lactate transport using AZD reduced glucose consumption and abrogated CL-mediated increased glucose uptake (Fig. 5, *D*–*E*). However, the contribution of glucose for the production of lactate was equal in all conditions. This was shown by isotopic studies in which we measured by NMR the labeling patterns of lactate produced by cells incubated with U-^13^C-glucose (Fig. 5*F*). We found that approximately 80% of lactate was derived from exogenous glucose, whatever CL or AZD treatments (Fig. 5*G*). Together, these experiments indicate that inhibiting lactate transport did not change the fraction of glucose contributing to lactate production but significantly impaired the glycolytic flux and reveal the importance of MCT1-dependent lactate fluxes for efficient utilization of glucose by beige adipocytes activated by the canonical β3-adrenergic signaling pathway.

### Concomitantly to lactate export, lactate import through MCT1 feeds the oxidative metabolism of beige adipocytes

**Figure 6:**
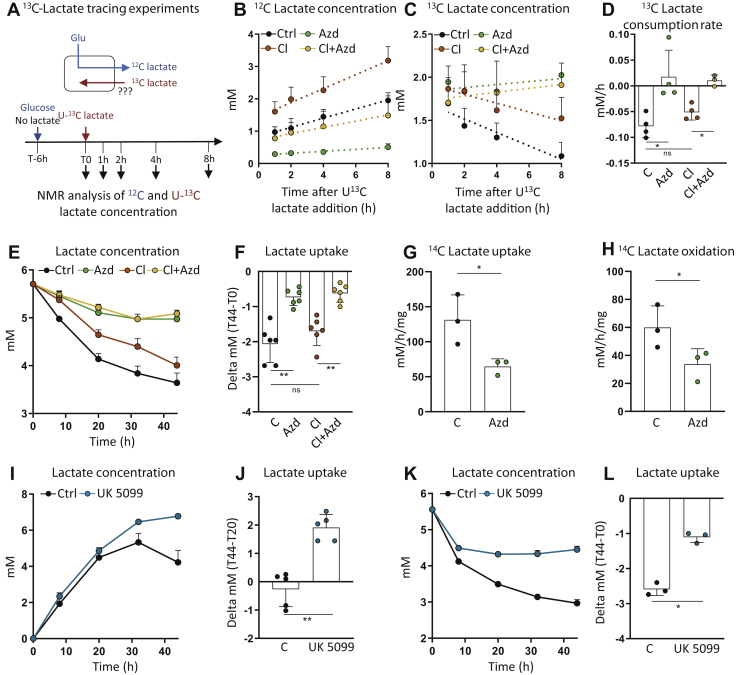
**Concomitantly to lactate export, lactate import through MCT1 feeds the oxidative metabolism of beige adipocytes.***A*–*D*, adipocytes were incubated in normal medium for 6 h, during which lactate was produced, before U-^13^C lactate was added to the medium. The concentrations of ^12^C (*B*) and ^13^C lactate (*C*) in cell supernatants were analyzed by NMR for 1, 2, 4, and 8 h after U-^13^C lactate pulse. The dotted lines represent linear regressions. *D*, ^13^C lactate consumption rate (*B*–*D*) (n = 4 independent primary cultures). *E*, time-course variation of lactate concentration in the supernatant of primary inguinal differentiated adipocytes treated with 50 nM AZD3965 (AZD), 10 nM CL316.243 (Cl), or both, in a lactate (5.5 mM), no glucose medium. *F*, net consumption of lactate between 0 and 44 h of culture by primary inguinal differentiated adipocytes treated with 50 nM AZD3965 (AZD), 10 nM CL316.243 (Cl), or both. (n = 6 independent primary cultures). *G* and *H*, ^14^C-lactate uptake (*G*) and oxidation (*H*) by primary inguinal differentiated adipocytes treated with 50 nM AZD3965 (AZD) (n = 3 independent primary cultures). *I*, time-course variation of lactate concentration in the supernatant of primary inguinal differentiated adipocytes treated with 50 μM UK5099, in a glucose-containing medium. *J*, net consumption of lactate between 20 and 44 h of culture by primary inguinal differentiated adipocytes treated with 50 μM UK5099, in a glucose-containing medium. (n = 5 independent primary cultures). *K*, time-course variation of lactate concentration in the supernatant of primary inguinal differentiated adipocytes treated with 50 μM UK5099, in a lactate (5.5 mM), no glucose medium. *L*, net consumption of lactate between 0 and 44 h of culture by primary inguinal differentiated adipocytes treated with 50 μM UK5099. (n = 3 independent primary cultures). Data are represented as mean ± SD. One and two-tailed nonparametric Mann–Whitney tests. ∗*p* < 0.05, ∗∗*p* < 0.01, and ∗∗∗*p* < 0.001.

As lactate net consumption was observed after 32 h of culture (Fig. 5*A*) when glucose concentration in the supernatant was very low (0.84 mM in control cells; Fig. 5*D*), we asked whether lactate import also occur in conditions where glucose is not limiting. To answer this question, cells were cultivated for 6 h in fresh medium, and then U-^13^C lactate was added to the medium. The concentration of labeled lactate added to the medium, *i.e.*, 2 mM, was chosen to minimize the addition of lactate while adding enough labeled lactate to measure the influx. The evolution of both ^12^C and ^13^C lactate concentrations in the medium was monitored from the NMR analysis of samples collected at different time points of the cultivation. As expected, a net production of lactate was observed (Fig. 6*B*), consistent with the glycolytic conversion of glucose into lactate. Very interestingly, a consumption of ^13^C lactate was observed in the same time (Fig. 6*C*), indicating that both export and import (release and consumption) of lactate occurred at the same time. The rate of ^13^C lactate import was significant (0.078 mM/h) and represented 55.19% of the export rate (0.141 mM/h). The import rate was slightly lower upon CL treatment but remained significant (Fig. 6, *C*–*D*). Both lactate release and consumption were strongly reduced upon MCT1 inhibition (Fig. 6, *B*–*D*). The strong lactate-consuming capacities of primary adipocytes and the role of MCT1 in lactate import were confirmed from the incubation of cells in a medium containing lactate but no glucose (Fig. 6, *E*–*F*). Incubation of adipocytes with ^14^C lactate confirmed the MCT1-dependent lactate uptake and showed that lactate is oxidized into CO_2_ through Krebs cycle activity (Fig. 6, *G*–*H*). To further study the link between lactate and oxidative metabolism, we performed experiments in which the mitochondrial pyruvate transport was pharmacologically inhibited using the UK5099 inhibitor. Very interestingly, we found that UK5099 abrogated lactate import, in both glucose-containing (Fig. 6, *I*–*J*) and lactate-containing (Fig. 6, *K*–*L*) media, demonstrating that lactate consumption is directly dependent on the mitochondrial pyruvate utilization. Together, these data highlight that MCT1 drives both the export and import of lactate and that the imported lactate feeds the oxidative metabolism of beige adipocytes.

## Discussion

**Figure 7:**
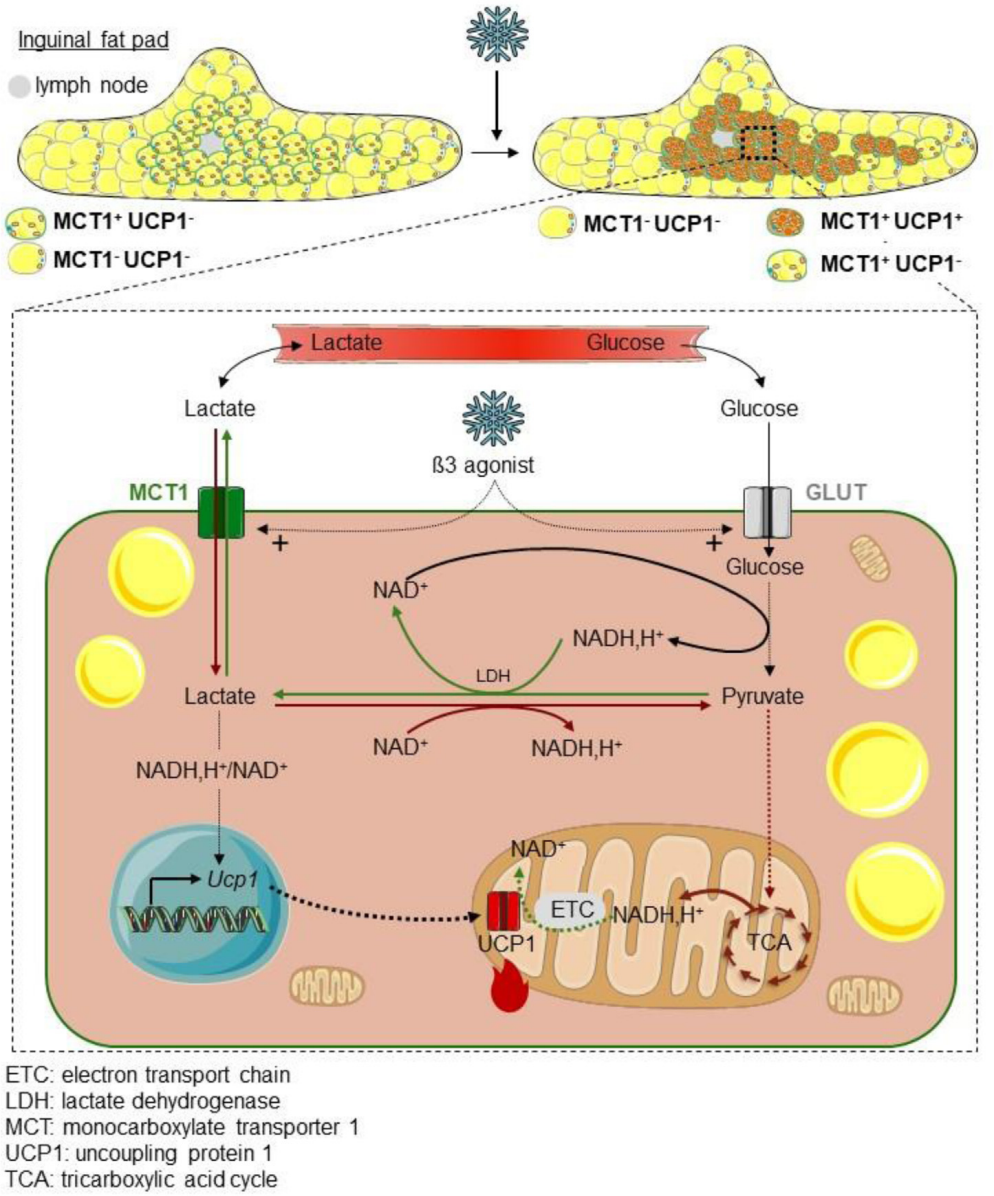
**MCT1-dependent lactate fluxes finely tune the metabolic activity of beige adipocytes.** This study highlighted MCT1 as a marker of cold-inducible beige adipocytes that mediates concomitant lactate export and import fluxes, which regulate glucose consumption and oxidative metabolism of beige adipocytes. ETC, electron transport chain; LDH, lactate dehydrogenase; MCT1, monocarboxylate transporter 1; TCA, tricarboxylic acid cycle; UCP1, uncoupling protein 1.

In the present work, we demonstrate that MCT1 is expressed by inducible beige adipocytes scattered in white adipose tissues and mediates bidirectional lactate transport, a process that finely tunes their metabolic activation by the β3 adrenergic system upon cold exposure (Fig. 7).

### MCT1 as a marker of inducible beige adipocytes

To date, there has been no report of molecular markers predictive of the susceptibility of white adipocytes to undergo beiging. In this work, we provide strong evidence that MCT1 expression is tightly correlated to the subpopulation of adipocytes susceptible to cold-induced beiging remodeling. The localization of MCT1^+^ adipocytes in the region of the inguinal fat pad prone to browning, their paucilocular phenotype, their mitochondrial enrichment, the positive correlation between *Mct1* and thermogenic gene expression at 21 °C, and the appearance of the UCP1 protein only in MCT1-expressing adipocytes upon 4 °C exposure together support the conclusion that MCT1 is expressed by adipocytes able to quickly initiate the thermogenic program and to express the UCP1 protein in response to cold. Whether the MCT1^+^ adipocytes present at 21 or 28 °C correspond to white adipocytes undergoing transdifferentiation or dormant adipocytes belonging to the beige lineage remains to be determined, these two hypotheses being debated (2, 11, 42, 43). The exclusive appearance of UCP1 in MCT1^+^ adipocytes from the beiging-sensitive inguinal adipose tissue is highly consistent with the lack of MCT1 protein in adipocytes from the perigonadal adipose tissue that is refractory to cold-induced UCP1 expression. Very interestingly, a study investigating cellular mechanisms occurring during postnatal development of BAT in Syrian hamster demonstrated that only adipocytes expressing MCT1 and containing middle-sized lipid droplets gave rise to mature UCP1^+^ brown adipocytes during the first days after birth (32), suggesting the existence of similar cellular processes between beiging and the development of brown adipocytes, in which MCT1 expression precedes UCP1 expression.

### Lactate transport finely tunes the metabolic activity of beige adipocytes

In addition to identify MCT1 as a marker of inducible beige adipocytes, the fine characterization of lactate fluxes notably through isotopic studies enabled us to show the existence of concomitant lactate export and import fluxes in beige adipocytes, which are both mediated by MCT1. This is consistent with the bidirectional transport mediated by MCTs, which depends on the electrochemical gradient of monocarboxylates and protons (26, 27). We demonstrated that MCT1-dependent lactate fluxes are required for the efficient utilization of glucose by beige adipocytes, which was not yet reported in this cell type, although glucose metabolism is largely known to support the metabolic activity of brown/beige adipocytes (44, 45, 46, 47). We also demonstrated that MCT1 is a transcriptional target of the β3-adrenergic pathway, further highlighting its role in adipocytes upon cold condition. The feedback inhibition on glycolysis when lactate export is impaired has been also reported in cancer cells (48, 49, 50) and probably occurs through a redox-dependent mechanism. Indeed, the conversion of pyruvate into lactate by lactate dehydrogenase is critical for the regeneration of NADH into NAD^+^, the mandatory coenzyme for the glycolytic glyceraldehyde dehydrogenase activity. When the lactate transport is blocked, lactate accumulates intracellularly, and the lactate dehydrogenase reaction slows down, resulting in lower NAD^+^ regeneration and high NADH/NAD^+^ ratio. This alteration in redox homeostasis can lead to cell death (48). The importance of redox homeostasis for brown adipocytes has been recently highlighted by the identification of Aifm2 oxidase, involved in NAD^+^ regeneration and glycolytic activity of BAT (51). Our data also show that MCT1-dependent lactate import is significant even in conditions where glucose is not limiting, further highlighting the important contribution of lactate inward flux to the biology of beige adipocytes including the regulation of *Ucp1* expression. We also demonstrated that the entering lactate can feed the oxidative metabolism of beige adipocytes. The significant influence of lactate on oxidative metabolism is consistent with its effects on mitochondrial uncoupled respiration (20). We also demonstrated that lactate consumption depends on the mitochondrial utilization of pyruvate. As the reaction catalyzed by lactate dehydrogenase is governed by the law of mass action, our results suggest that the mitochondrial utilization of pyruvate pulls the lactate dehydrogenase reaction toward lactate utilization, and hence favors lactate import. The importance of lactate as an oxidative substrate has been recently highlighted (52). The β3 adrenergic system stimulated both glycolysis and lactate production but did not increase lactate consumption rate. As MCT1 was not involved in the signaling effect of the β3 adrenergic system on *Ucp1* expression, it therefore appears that MCT1-dependent lactate fluxes are key for cold-induced glucose consumption by beige adipocytes but not for cold-induced signaling effects on *Ucp1* expression. Further investigations are therefore definitively needed to decipher the physiological and pathophysiological situations stimulating net lactate consumption by beige (and brown) adipose tissues.

The bidirectional exchange through MCT1 participates to equilibrating the internal and external lactate pools. As a result, the level of exogenous lactate is directly impacting the internal concentration of lactate, hence the equilibrium of the lactate dehydrogenase reaction, the NADH/NAD^+^ ratio, and thus the glycolytic activity. This process may represent an efficient mechanism for sensing extracellular lactate levels and fine tuning the metabolic activity of beige adipocytes. Because beige adipocytes strongly express MCT1 and exhibit intense metabolic activity, one can also speculate that they could play a key role in dissipating high redox pressure in their microenvironment (17, 23). In conclusion, we propose that MCT1, by enabling an efficient exchange of lactate across the plasma membrane, tightly connects extracellular metabolic conditions to the intracellular metabolic activity of beige adipocytes and that lactate fluxes are key regulators of the metabolic activity in beige adipocytes.

## Experimental procedures

### Animal experiments

Animal studies were carried out using 6-week-old male C57BL/6J mice obtained from the Envigo Laboratory. All animals were housed with three to five mice per cage in a controlled environment on a 12 h light/dark cycle with unrestricted access to water and a standard chow diet in a pathogen-free animal facility. All studies were carried out under the Institut National de la Santé et de la Recherche Médicale (INSERM ) Animal Care Facility guidelines and local ethical approval by the Institutional Animal Care and Use Committee of Région Midi-Pyrénées (France). Mice were maintained in temperature-controlled room at 21 °C or exposed to 28 °C (10 days) or 4 °C (24 h, 48 h, or 7 days). Mice were killed by cervical dislocation, tissues were dissected and processed for histology or immediately flash-frozen in liquid N_2_, and stored at −80 °C until further analyses. All experimental procedures were done in compliance with French Ministry of Agriculture regulations for animal experimentation.

### Primary culture of adipose-derived stem/stromal cells and adipocyte differentiation

Inguinal adipose tissue excised from mice were finely cut out and digested at 37 °C in PBS containing collagenase NB4 (standard grade from Coger) 0.4 U/ml for 30 min. After elimination by filtration through 25 μm filters of undigested fragments, mature adipocytes were separated from pellets of stromal vascular fraction cells by centrifugation (1800 rpm; 10 min). After the lysis of red blood cells, stromal vascular fraction cells were resuspended in complete culture medium (minimum essential medium alpha plus 0.25 units/ml amphotericin, 100 units/ml penicillin, 100 mg/ml streptomycin, biotin 0.016 mM, ascorbic acid 100 μM, panthotenic acid 0.018 mM, and 10% newborn calf serum), counted, plated at 10,000 cells/cm^2^, and then rinsed in PBS 5 h after plating. Cells were maintained at 37 °C (5% CO_2_) and refed every 48 h. Adherent adipose-derived stem/stromal cells were grown to confluence and exposed to the adipogenic cocktail containing 5 μg/ml insulin, 2 ng/ml T3, 33.3 nM dexamethazone, 10 μg/ml transferrin, and 0.1 μM rosiglitazone in complete medium. Adipocytes differentiated for 5 days were treated with different compounds at the time and concentrations as indicated in the legends to the figures.

### ^14^C lactate uptake and oxidation assay

Differentiated adipocytes were incubated with L-[U-^14^C]-lactate (1 μCi/ml) and nonlabeled (cold) lactate (1 mM) for 3 h. Following incubation, ^14^C lactate in the sample and ^14^C CO_2_ released in the medium were measured as previously described (53). Briefly, assayed medium was transferred into a custom-made Teflon 48-well trapping plate. The plate was clamped and sealed, and perchloric acid was injected through perforations in the lid into the medium, which drives CO_2_ through a tunnel to an adjacent well, where it was trapped in 1 N NaOH. Following trapping, aliquots of NaOH were transferred in scintillation vials, and radioactivity was measured with a multipurpose scintillation counter (LS 6500; Beckman Coulter). Data are normalized to cell protein content.

### ^13^C stable isotope tracing experiments

For ^13^C lactate tracing experiments, primary adipocytes differentiated for 5 days in 56.7 cm^2^ cell culture dishes were cultivated in defined medium (αMEM Gibco ME16265L1) containing 5% dialyzed serum and 0.5 mM glutamate, 2 mM glutamine, 1 mM pyruvate, and 5.5 mM glucose, supplemented with all the molecules of the complete medium described previously. After 6 h of culture, 2 mM U-^13^C lactate was added, and 300 μl of culture medium was collected for NMR analysis after 1, 2, 4, and 8 h.

For ^13^C glucose tracing experiments, cells were incubated with the same medium, but unlabeled glucose was replaced by U-^13^C glucose (5.5 mM). Culture medium was collected for NMR analysis after 24 h.

### NMR spectroscopy experiments

The labeling patterns of medium metabolites in ^13^C-labeling experiments were measured by 1D ^1^H- and 2D heteronuclear J-resolved spectroscopy NMR spectroscopy (54). All NMR spectra were recorded on a Bruker Avance III 800 MHz spectrometer (Bruker Biospin, Rheinstetten, Germany) equipped with a quadruple resonance inverse cryoprobe 5 mm cryogenic probe head. Spectra were acquired and processed using the Bruker Topspin 4.0 software (Bruker Biospin, Rheinstetten, Germany). Analyses were performed at 280 K. Acquisition conditions for 1D ^1^H-NMR spectra were as follows: 30° flip angle, 5000 Hz spectral width, 32 K memory size, and 30 s total recycle time. The 2D zero quantum filtered-H-JRES spectra were recorded with quadrature phase detection in both dimensions, using time-proportional phase incrementation in the indirect dimension, 128 increments in the F1 dimension, and 8 transients per increment, and were accumulated with the same sweep width and acquisition times as in 1D experiments. The specific enrichment in ^13^C of metabolites was measured from the J_CH_ coupling signals in the ^1^H-NMR spectra (54). Because of partial overlap with other signals, the quantification of unlabeled and labeled lactate was performed by deconvolution of the ^1^H signals corresponding to ^13^C-uncoupled and ^13^C-coupled H3 protons, respectively.

### RNA extraction and real-time PCR

Total RNA from cells was isolated and extracted using the ZYMO RNA kit (Zymo). For mouse tissues, total RNA was isolated by Qiazol extraction, and purification was done using RNeasy minicolumns (Qiagen). For quantitative real-time PCR analysis, 300 to 1000 ng total RNA was reverse transcribed using the High Capacity cDNA Reverse Transcription kit (Life Technologies/Applied Biosystem), SYBR Green PCR Master Mix (Life Technologies/Applied Biosystem), and 300 nmol/L primers on an Applied Biosystem StepOne instrument. Primers are listed in Table 1. Relative gene expression was determined using the 2^−ΔCT^ or 2^−ΔΔCT^ method as described in the legends to the figures and normalized to *36B4*.

**Table 1:** Primer sequences

Gene	Primer reverse	Primer forward
*36B4*	AGTCGGAGGAATCAGATGAGGAT	GGCTGACTTGGTTGCTTTGG
*Ucp1*	GACCGACGGCCTTTTTCAA	AAAGCACACAAACATGATGACGTT
*Cox8b*	GAACCATGAAGCCAACGACT	GCGAAGTTCACAGTGGTTCC
*Cidea*	CTAGCACCAAAGGCTGGTTC	CACGCAGTTCCCACACACTC
*Mct1*	TGTGGGCTTGGTGACCAT	AAGAGATAGATACCCGCGATGATG
*Mct2*	CACCACCTCCAGTCAGATCG	CTCCCACTATCACCACAGGC
*Mct3*	CGCTTCCCTAGTGCATTGG	TTCTTCAGAGCATCCACCAG
*Mct4*	AGTGCCATTGGTCTCGTG	CATACTTGTTAAACTTTGGTTGCATC
*Ob*	ACCACCATTGTCACCAGGATCAA	ACCCTCTGCTTGGCGGATA
*Acc*	GACTTGCAGAAGAAATACGCCATA	CTTGTATCCCTTGTAGGGATCTTC
*Cd36*	GATGTGGAACCCATAACTGGATTCAC	GGTCCCAGTCTCATTTAGCCACAGTA
*Ap2*	GATGCCTTTGTGGGAACCTG	GCCATGCCTGCCACTTTC
*Pparg2*	AGTGTGAATTACAGCAAATCTCTGTTTT	GCACCATGCTCTGGGTCAA

### Lactate and glucose measurements

Extracellular lactate levels were measured using the Lactate Pro II test meter (Arkray). Glucose concentrations in adipocyte supernatants were measured using Contour XT TS (Bayer).

### LCM

Inguinal fat pad harvested from fed mice was fixed in methanol (−20 °C, overnight) and placed in a plastic cryomold filled with tissue freezing compound (Tissue-Tek OCT), frozen in isopentane, and stored at −80 °C before being sliced into 50 μm sections using a cryostat (MICROM HM 560V). Sections were placed on membrane-coated slides, immersed in chilled 70% ethanol for 30 s, and then rinsed in water for 15 s. Slides were then immediately immersed in graded series of ethanol (70, 95, and 100%, for 30 s). Tissue sections were cleared in xylene for 2 min and air dried for 5 min. All steps were performed in RNAse-free conditions. LCM was carried out under 10× magnification microscope using the ARCTURUS XT apparatus (ArcturusXTTM microscope system). Dissected cells were collected in the collecting tube cap filled with 50 μl of lysis buffer (Arcturus PicoPure RNA isolation Kit). Total RNA was extracted according to the manufacturer's instructions. About 30 ng of total RNA was reverse transcribed using the Superscript Vilo cDNA synthesis kit (Life Technologies). A multiplex PCR preamplification (12 cycles) of the specific cDNA targets was performed using GE Preamp master mix (Fluidigm). About 2 μl of the diluted 1/50^e^ preamp reaction was used for quantitative real-time PCR as described.

### Immunofluorescence and quantification

Inguinal, interscapular BAT, and perigonadal white adipose tissues were fixed in 4% paraformaldehyde overnight before being cut into 300 μm sections using a vibratome (Campden). Sections were incubated in blocking solution (2% normal horse serum and 0.2% triton X-100 in PBS) at room temperature 6 h before being incubated for 24 h at room temperature with primary antibodies (sheep anti-UCP1 1:1000 generous gift from Pr. Daniel Ricquier, rabbit anti-MCT1 1:200 EMD Millipore AB3538P, chicken anti-MCT1 1:500 EMD Millipore AB1286I, and rabbit TOM20 1:400, Santa Cruz, sc-11415). After overnight incubation at 4 °C with Alexa488- or Alexa555-conjugated secondary antibodies (Life Technologies) and 30 min with 4',6-diamidino-2-phenylindole (1:10,000), imaging was performed using a confocal laser scanning microscope (LSM880; Carl Zeiss) and image analysis using Fiji software (National Institutes of Health). For quantification of UCP1 and MCT1 signals, manually drawn regions of interest were performed around cells using autofluorescence and MCT1 plasma membrane signals and were reported on UCP1 staining images after splitting MCT1 and UCP1 channels of coimmunostained adipose tissue sections. For each signal and each region of interest, mean gray values, area, and coordinates of ROIs were measured. The threshold of mean gray value for positive cells was manually fixed. Four cell populations were thus quantified (MCT1^+^ UCP1^−^, MCT1^+^ UCP1^+^, MCT1^−^ UCP1^−^, and MCT1^−^ UCP1^+^).

### Statistical analysis

Data are expressed as mean ± SD. Graphic representation of data as well as all statistical tests were performed with GraphPad Prism. Comparisons among different groups were analyzed with nonparametric Mann–Whitney test. Statistics on simple linear regressions were performed with Pearson's test. The number of independent experiments and n values are specified in the legends to the figures. Differences were considered statistically significant at *p* ≤ 0.05.

## Data availability

All data for this publication are included in this published article.

## Conflict of interest

The authors declare that they have no conflicts of interest with the contents of this article.
